# Food texture experiences across nine age groups in Indian infants from urban areas

**DOI:** 10.3389/fnut.2024.1419718

**Published:** 2024-07-18

**Authors:** Marine Devezeaux de Lavergne, Frank Thielecke, Nicolas Antille, Lisa R. Fries, Carolyn F. Ross, Sarah Smith-Simpson

**Affiliations:** ^1^Nestlé Institute of Food Sciences, Nestlé Research, Société des Produits Nestlé S.A., Lausanne, Switzerland; ^2^Department of Health Promotion, Swiss Distance University of Applied Sciences, Brig, Switzerland; ^3^T2 Bene Ltd., Allschwil, Switzerland; ^4^Nestlé Institute of Health Sciences, Nestlé Research, Beijing, China; ^5^School of Food Science, Washington State University, Pullman, WA, United States; ^6^Nestle Nutrition, Société des Produits Nestlé S.A., Fremont, CA, United States

**Keywords:** complementary food, food texture introduction, texture acceptance, young children, food diversification, parental survey

## Abstract

The introduction of complementary food plays a fundamental role in dietary behaviours later in life. Little is known about the influences of age on food texture acceptance in young Indian children. Thus, the objective of this cross-sectional study was to describe the relationship between age and food texture experiences in young children aged 4–36 months in India from urban areas using a parental-reported survey. This study relies on a face-to-face parent survey, which was conducted comprising 306 children categorised into 9 age groups. Questions focussed on food texture experience considering 16 textures were analysed. Textures such as *dissolvable*, *sticky*, and *soupy*/*liquidy* were already accepted by more than half of 4–5-month-old infants. In India, *soupy*/*liquidy* is a more common base texture than *pureed*. Indeed, *pureed* was found to be introduced to a majority of infants only from 8 to 9 months onwards. Food textures such as *rubbery*, *slippery*, and *foods with skin* were more likely rejected by the youngest children. With increasing age, the refusal probability of food textures decreased. Our survey showed food texture experiences in Indian children aged from 4 to 36 months. It provides useful insights for parents and healthcare professionals by contributing to the understanding of texture acceptance during the transition to complementary foods.

## Introduction

Complementary feeding describes how infants gradually transition from drinking only milk to consuming a whole range of foods (1). It is widely accepted that the introduction of complementary food plays a fundamental role in an infant’s developmental progress as well as dietary behaviours later in life (2, 3). While guidelines exist around the globe on when to start complementary feeding (e.g., from the WHO), specific guidelines regarding when to introduce infants to certain food textures are often confined to the introduction of *pureed* or *mashed* foods at the age of 6 months (4, 5). The European Food Safety Authority states that the developmental skills required to consume complementary foods will differ depending on the texture of the food (6). The Indian Ministry of Health and Family Welfare recommends introducing *lumpy* foods from 6 months of age onwards and progressing to *finely chopped foods* that require chewing from 9 months old (7).

“Texture” is a multidimensional sensory property that usually describes several sensory variables and depends upon the mechanical and geometrical properties of the food (8, 9). Moreover, texture changes during the oral processing of the food and different texture attributes might appear at different time points of consumption (10), which reflects the complexity of texture definition and measurement. Current evidence suggests that early introduction of new food textures supports infants to accept new textures thereafter and to progress with new texture acceptance (3). Studies show that the timing of texture introduction to infants’ diets can be important for improved eating behaviours and later texture acceptance. For instance, children who received lumpy foods before the age of 6 months were less “fussy” and ate more fruits and vegetables than those who received lumpy foods after 10 months (11). It can be problematic that textures in surveys are often illustrated using several example foods (12), which frequently results in loss of detail about specific textures. For example, the food category “vegetables” in one study contains 49 food texture combinations in total (13). The same authors categorised food texture combinations into three texture levels: texture level 1 “*purees*,” (*soft and rough*), texture level 2 “*small*/*soft pieces*,” and texture level 3 “*hard*/*big pieces and double texture*.” This illustrates that details of specific textures are easily lost, and only “texture levels” can be related to other factors (1, 14). Furthermore, the categorisation of textures in food is not only subjective but also complicated by the fact that food rarely possesses just one texture, of which the several textures reported may depend upon the preferences of the participant. Therefore, this approach may not capture the various experiences of participants. It may also not account for potential cultural variability.

It is known that exposure to complex food textures early in life supports the development of oral motor skills. A wide panel of textures introduced to the diet allows infants to develop important oral motor skills such as tongue lateralisation (15). Furthermore, promoting food variety in the sense of texture and taste, especially with an age of 9–12 months, aids the development of mastication and the adoption of foods with more complex textures and tastes (16). It has been proposed that early exposure to a variety of textures aids in the development of infants’ oral motor skills. Specifically, introducing larger and harder food pieces around the age of 8 months may positively influence infants’ chewing abilities and their acceptance of different textures (17). While the link between the development of infant oral motor skills and the ability to manage textures of increasing complexity has been subject to recent research, little information is available on the introduction of complex textures such as *leafy*, *chewy*, *hard*, or *combination textures*. The American Academy of Pediatrics has outlined milestones for food texture introduction to ensure that children are safely introduced to a variety of textures (18). To date, most data on age and food texture introduction are from France and the United States (1, 14, 19, 20). Moreover, current surveys on the influence of age on texture introduction are limited in the number of textures, including *pureed*, *soft small pieces*, *hard*/*large*, a *combination of textures*, *lumpy*, and *dissolvable* (13, 21, 22). The study by Surette et al. (19) proposed 16 textures, which were assessed in five age groups (AGs). As parents have been observed to report food items rather than texture attributes when asked about introduction of food textures (24), Surette et al. (19) proposed a list of 16 textures illustrated by a list of food items typically associated with this texture attribute, while covering a variety of taste and flavour to avoid associations between texture and other food item features. The textures selected cover a wide range of textures that young children would commonly eat, combining attributes from several studies. This list covers crispy, chewy, creamy, dissolvable, hard, juicy, lumpy, pureed, rubbery, slippery, soft, sticky, and tough meat [from Ross et al. (24)] and combination textures [from Demonteil et al. (1)]. However, this list required an adaptation to the cultural context of texture introduction outside of the United States. In our survey, we used the same method as Surette et al. (22) which considers the role of age on food texture introduction and food texture acceptance in children.

Refusal of food textures is another relevant variable in food texture experience (25, 26). Several studies in healthy children report food refusal based on texture at a magnitude of 36.2% (27), and in children with intellectual disabilities up to 72% (28). Food refusal in preschool children due to refusal to chew or high food temperatures has been reported (29). Few studies, predominantly from the United States and France, have explored child behaviours, such as the acceptance of foods based on food texture (1, 14, 19). It is not necessarily possible to generalise these findings to other countries and cultures. In fact, little is known about cross-cultural differences in texture introduction practices. Specific data from India on this matter are scarce; however, a recent study reported the introduction of a range of *soft*, *minced* and *easy-to-chew* food among infants aged 6–9 months (30). Moreover, data from the National Family Health Survey in India reveal that a low dietary diversity is associated with an increased risk of undesirable health outcomes and calls for more research on complementary feeding practices in India (31).

To our knowledge, no information on texture acceptance in young children is available in India. This study aimed to extend the understanding of the role of age on texture acceptance and refusal in a population of Indian children aged 4–36 months from urban areas. Taking cultural practices of texture introduction into account is an important part of this study. As infants’ oral motor skills develop quickly over the first year of life (32, 33), analysing food texture experience in nine AGs, of 4–5, 6–7, 8–9, 10–11, 12–14, 15–17, 18–23, 24–30, and 31–36 months, as compared to five AGs provides more detail in this period of rapid development. Similar narrow intervals of approximately 2 months up to the first year of age when introducing food textures have been postulated by others (34). Given the sparseness of studies evaluating refusal of food textures in young children, exploration of refusal in the 16 food textures is also included in our study.

## Methodology

### Participants

For this survey, 306 families were recruited through a consumer research agency from two centres. One centre consisting of 155 families was located in the North of India, in the Delhi metropolitan area (population of approximately 22.5 million people). The other centre, consisting of 151 families in the West of India, is in the Kolkata metropolitan area (population of approximately 19.4 million people) (35). Each centre was divided into four zones (North, South, East, and West). Participants were initially identified based on the Indian electoral register to ensure the capture of all zones across the metropolitan areas. In each zone, a starting address was selected at random, and subsequent addresses were identified using structured intervals; i.e., households were approached using the “right-hand rule.” This rule entailed that the interviewers moved to the next house on the right from the starting address. In case a household refused to participate or was deemed ineligible, interviewers moved to the immediate next house on the right. After a successful interview, two houses were skipped, and interviewers then approached the third house on the right.

The inclusion criteria for the current study were as follows: participants had at least one child between the ages of 4 and 36 months who had already begun consuming solid foods. Families with more than one eligible child were asked to complete the survey study for the youngest child within the target age range. In this study, the primary caregivers were interviewed.

The children were categorised into nine AGs of 4–5, 6–7, 8–9, 10–11, 12–14, 15–17, 18–23, 24–30, and 31–36 months. The AGs were selected based on child feeding studies (1, 26, 36) as well as a reflection of the substantial changes in texture introduction and acceptance over this short period in recent research (19). These nine AGs were then used to analyse the role of age on texture introduction. As the evaluation of texture management and refusal only included infants who had already been exposed to that texture, AGs 1 and 2 were combined. This approach reflects the relatively low introduction rate of textures at these ages and ensures a sufficient sample size for statistical analysis. A similar methodology has been described elsewhere (19). Merging these two age groups still allows for drawing conclusions relevant to younger age groups.

### Survey

Prior to the actual study, a pilot survey was conducted with 10 parents to assess the applicability of the survey in an Indian context, i.e., to ensure all questions were understandable including the texture categories and food examples given and to understand the accessibility to food items. Data were collected between March and April 2023 using a structured questionnaire. The survey was based on a previously developed online questionnaire (19, 37). In detail, the texture selections of the survey were derived from established literature. Ross et al. (24) reviewed survey responses from parents of children with Down’s syndrome, who indicated which textures were “easy” or “difficult.” They found that parents frequently named food items rather than specific textures. For our research, we included 13 textures. Additionally, combination textures were sourced from Demonteil et al. (1), and other common textures for young children and were included based on Szczesniak (38) and Welker et al. (39) resulting in a total of 16 textures for this survey (crispy, chewy, creamy, dissolvable, hard, juicy, leavy, lumpy, pureed, rubbery, slippery, soft, sticky, foods with skin, combination textures, and soupy/liquidy). Food examples were matched to each texture, drawing from various studies to ensure relevance and clarity. In the present study, trained personnel conducted face-to-face interviews with the parent responsible for feeding the child. The experienced interviewers received additional training and familiarised themselves with the specific survey during the pilot study.

Our parent-reported survey consisted of 35 questions, mainly focussing on the children’s food texture experiences, as well as demographic questions about birthdate, sex, whether breastfed, duration of breastfeeding, and at what age they first served their child complementary foods (defined as any food other than breast milk or formula). Participants were also asked about their child’s teething status, including the age when teething started and if their child was currently teething at the time of the study.

Details of the questionnaire have been described elsewhere (19, 37). After answering the demographic questions, participants were presented with the definition of texture, defined as “the feel, appearance, or consistency of a surface or a substance.”

For the present study, three texture-associated questions were applied to a list of 16 textures. Two changes were made from the texture list used in the previous study in the United States (19): the category of *tough meat* was removed, and a new texture of *soupy*/*liquidy* was added. These changes were culturally driven, based on insights from local experts and given the circumstances of the Indian complementary food culture. To ensure that participants understood the texture descriptors, food examples with varying taste profiles were provided for each texture (Table 1).

**Table 1:** Food textures with corresponding food examples.

Textures	Examples
Crispy foods	Potato chips, wafer chocolates, papad, crackers (crispy biscuits)
Chewy foods	Raisins, roti, paratha, luchi/poori, grilled chicken
Creamy foods	Yoghurt, custard, mishti doi
Dissolvable foods	Rice puffs, glucose biscuits
Hard foods	Nuts and lollipops, peanut chikki, carrot sticks
Juicy foods	Oranges, tomatoes, watermelon
Leafy foods	Salad greens, methi, amaranth leaves bhaji
Lumpy foods	Ready-to-eat baby food/cereal mashed potatoes, dalia, sooji halwa
Pureed foods	Stewed apple, carrot puree, vegetable puree, aamras
Rubbery foods	Mushrooms, cheese slice/cubes, burger, rasgulla
Slippery foods	Noodles, hard-boiled eggs, mango, kiwi, bhindi (okra)
Soft foods	Bananas, besan chilla, khichuri, fish
Sticky foods	Sticky foods, such as honey, dry fruit chutney
Foods with skin	Grapes, apples, guava, whole pulses, sprouts
Foods with a combination of textures	Vegetable khichdi, dal chawal, vegetable sandwich, poha
Soupy/liquidy texture	Dal water, buttermilk (ghol), clear chicken soup

The main focus of this study is reflected in three questions from the survey relating to food texture experience:

*Question 1*: “*Tell us if your child has tried this food texture*.”

Possible responses were (1) not yet; (2) yes, but only once; and (3) yes, multiple times. This 3-point scale was consolidated to a 2-point scale (“tried” and “not tried”). Responses were converted: from “not yet” to “not tried”; from both “yes, but only once” and “yes, multiple times” to “tried.”

*Question 2*: “*How well can your child manage [a previously tried] texture*?”

The response was based on the perceived difficulty of the food texture for the child to eat. Possible responses were on a 5-point Likert scale ranging from 1 = very difficult to 5 = very easy.

*Question 3*: “*In the past month*, *which of the following food textures has your child refused to eat*?”

Participants could check all applicable textures.

Furthermore, the approach to assess texture sensitivity in this survey was based on previous literature and included the following questions (22):

1. “My child limits self to certain textures.”
2. “My child is a picky eater, especially about food textures.”
3. “My child prefers one texture of food.”
4. “My child would rather drink than eat.”
5. “When you introduce new textures into your child’s diet, do you feel confident that he/she will accept these foods?”

Possible responses for questions 1–4 were on a 5-point scale: (1) almost never, (2) infrequently, (3) sometimes, (4) frequently and (5) almost always. Possible responses for question 5 were binary with (1) for “yes” and (0) for “no.” Answers from questions 1–4 were transformed to a binary score, with a (1) assigned to scores 5–4 and (0) assigned to scores 1–3; these recoded items and question 5 were summed and a total of 2 points or more resulted in classifying a child as texture sensitive (22).

Finally, a list of questions related to perceptions of food safety was administered.

This study was deemed IRB Exempt by the Washington State University Institutional Review Board (WSU IRB #17585), and a written consent was obtained from all study participants.

### Statistical analysis

#### Question 1

R 4.0.2 (23) was used to perform an ordered probit model and contrast separations for the consolidated 2-point scale on the tried-texture data; the independent variables were AG, sex, and texture sensitivity. Pairwise chi-squared tests were performed to compare the proportion of children having tried a texture across AGs. A 95% confidence level was applied for all statistical tests. The average number of food textures introduced to children within each AG was also calculated.

#### Question 2

SPSS v 28.0.1.1 was used to perform an analysis of variance, least-square means, and Tukey’s honestly significant difference mean separation on the texture management data; the independent variable was AG. Of note, AG1 and 2 were merged for the analysis of both texture management and texture refusal.

#### Questions 3

R 4.0.2 was used to perform an ordered probit model on the data of refused textures; the independent variable was AG. Finally, the average refusal proportion across all AGs was calculated and pairwise chi-squared tests were performed to compare these proportions across textures.

## Results

### Population

A total of 306 participants completed the study, with slightly more male children (*n* = 160) than female (*n* = 146). The participating families were from urban settings, with all respondents being mothers. Mothers residing in the Delhi metropolitan area generally had higher average educational levels than those in the Kolkata metropolitan area. However, the distribution of household income was similar across both regions (Supplementary Tables S1, S2).

Approximately 96% of the study cohort had a history of breastfeeding, either in the past or at present. More than 75% of the mothers were still breastfeeding at the time of the survey. The average age for introducing complementary foods was 6.3 ± 1.6 months overall, with a slightly earlier introduction observed in Delhi at 6.1 ± 1.6 months than Kolkata at 6.6 ± 1.5 months (*p* = 0.01). Demographic characteristics of the participants by AG, sex, teething, and texture sensitivity are presented in Table 2. Most children, 252 out of 306, were reported to have started teething at the time of the survey, and 207 out of 306 children were reported to be sensitive to texture, based on the 5-item questionnaire.

**Table 2:** Demographic characteristics of the study population (*N* = 306): individuals were stratified by age group, sex, distribution of teething status, and food texture sensitivity characterisation.

Age group	Age (months)	Sex		Started teething		Texture sensitive		Total
		Male	female	Yes	No	Not TS	TS	
1	4–5	11	10	19	2	6	15	21
2	6–7	10	12	19	3	6	16	22
3	8–9	13	15	22	6	7	21	28
4	10–11	11	10	17	4	7	14	21
5	12–14	24	14	28	10	11	27	38
6	15–17	7	12	17	2	9	10	19
7	18–23	42	26	58	10	20	48	68
8	24–30	29	32	53	8	20	41	61
9	31–36	13	15	19	9	13	15	28
Total		160	146	252	54	99	207	306

### Role of age on food texture introduction

**Figure 1:**
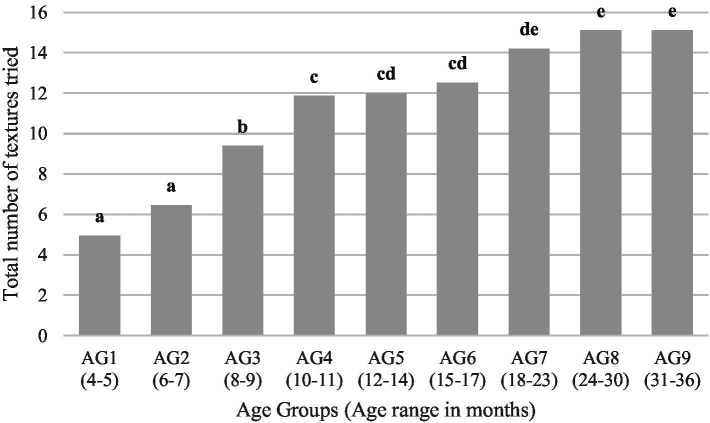
Numbers of textures tried by age group. Different letters indicate significant differences between age groups.

**Figure 2:**
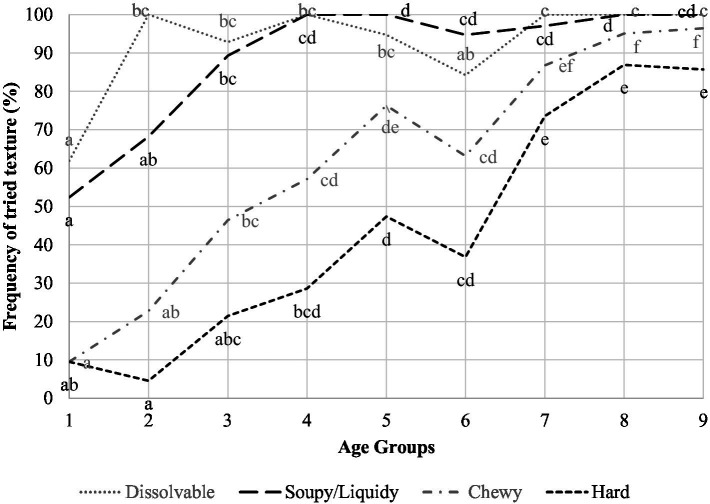
Introduction rate of chosen textures across all age groups for four of the textures Different letters indicate significant differences among the age groups for a given texture.

In 4% of the infants, the introduction of complementary foods started as early as 3 months. The most common age to start introducing complementary foods was 6 months (50% of infants), followed by 7 months (24%). Out of 16 texture categories presented, the average number of textures introduced by AG is shown in Figure 1. Significant increases in textures tried were from AG2 to AG3, and from AG3 to AG4. Furthermore, children tried significantly more textures in AG7 than AG4, and in AG8 and AG9 than AG5 and AG6. Sex and texture sensitivity had no significant effect on texture introduction. The introduction of food textures in relation to age is shown in detail in Table 3. For all textures, the prevalence of introduction increased with advancing AGs. Across all AGs, the introduction of the textures *sticky* and *dissolvable* was least influenced by age, whereas the introduction of textures such as *chewy*, *hard*, *leafy*, and *slippery* was most influenced by age. Correspondingly, with increasing AG, there was a concomitant increase in the variety of food textures introduced. The frequency of chosen textures is depicted in Figure 2 for a selection of four textures. D*issolvable* was more often tried in AG2 than AG1. *Soupy*/*liquidy* showed a significant increase in frequency from AG3 compared to AG1 and plateauing afterwards. *Chewy* and *hard* demonstrated a different pattern—a significant increase in the frequency from AG1 to AG3 for *chewy* and AG1 to AG5 for *hard*, both peaking at AG7, with a continued slight increase afterwards. Statistical analysis revealed that the variable “Started Teething” was highly correlated with the AG and was therefore not included as a separate variable.

**Table 3:** Impact of age, sex, and texture sensitivity characterisation of food texture introduction.

Food texture	Age group		Sex		Texture sensitive	
	*z*	*p* > |*z*|	*z*	*p* > |*z*|	*z*	*p* > |*z*|
Crispy	8.56	<0.001	1.14	0.256	−1.03	0.303
Chewy	9.27	<0.001	1.15	0.251	−0.27	0.785
Creamy	8.33	<0.001	0.47	0.641	1.74	0.082
Dissolvable	4.04	<0.001	−0.01	0.995	−0.31	0.757
Hard	9.41	<0.001	0.48	0.628	−0.18	0.860
Juicy	7.53	<0.001	1.15	0.248	0.60	0.551
Leafy	9.05	<0.001	1.07	0.287	−1.65	0.098
Lumpy	6.61	<0.001	0.26	0.792	0.69	0.487
Pureed	5.17	<0.001	−0.42	0.671	−0.49	0.628
Rubbery	8.51	<0.001	−0.21	0.834	0.37	0.712
Slippery	9.58	<0.001	0.14	0.886	−1.96	0.050
Soft	6.13	<0.001	0.90	0.368	0.36	0.720
Sticky	3.49	<0.001	−0.55	0.585	−0.67	0.505
Foods with skin	7.91	<0.001	0.30	0.766	0.65	0.514
Combination	6.60	<0.001	1.48	0.139	−0.05	0.963
Soupy/liquidy	5.36	<0.001	1.21	0.228	−0.06	0.956

The *z*-scores and *p*-values correspond to the model including all three variables. All variables were treated as continuous, included AG (numbered from 1 to 9). Sex and texture sensitive are binary variables. The positive *z*-scores for AG show that a texture is more likely to have been introduced if the child is older. As sex is coded with 1 = male and 2 = female, a positive *z*-score indicates that the texture was more frequently introduced to girls. However, the sex effect is never significant. As texture sensitive is coded with 1 = yes and 2 = no, a positive *z*-score indicates that the texture was more frequently introduced to children that are not texture sensitive.

Table 4 illustrates the distribution of children within each AG who tried each food texture. The prevalence of children trying *dissolvable*, *soft*, and *combination textures* showed a statistically significant increase from AG1 to AG2 and subsequently plateaued from AG2 (*dissolvable*) or AG4 (*soft* and *combination textures*). Textures such as *chewy*, *hard*, *juicy*, *lumpy*, *rubbery*, *slippery*, *foods with skin*, and *soupy*/*liquidy* increased significantly from AG1 to AG3 and continued the rise across the AGs. The percentage of children that tried *creamy*, *hard*, *leafy*, and *pureed* increased up to AG4 or AG5. For textures *hard*, *crispy*, *creamy*, and *leafy,* this trend continued to AG9. The percentage of children trying textures such as *pureed* and *sticky* increased up to AG4, with no significant changes thereafter. Most children tried the texture *dissolvable* in AG2, and textures like *pureed*, *soupy*/*liquidy*, and *lumpy* had been tried by most infants in AG4, whereas the highest proportion of children were observed trying the other textures in AG7–AG9.

**Table 4:** Parental responses to the question: “Tell us if your child has tried these food textures.”

Texture	% Tried per age group								
	AG1 (4–5 months)	AG2 (6–7 months)	AG3 (8–9 months)	AG4 (10–11 months)	AG5 (12–14 months)	AG6 (15–17 months)	AG7 (18–23 months)	AG8 (24–30 months)	AG9 (31–36 months)
Crispy	29^a^	32^a^	46^ab^	57^abc^	74^cd^	68^bcd^	87^de^	98^f^	96^ef^
Chewy	10^a^	23^ab^	46^bc^	57^cd^	76^de^	63^cd^	87^ef^	95^f^	96^f^
Creamy	24^a^	27^a^	50^ab^	67^bc^	76^c^	74^bc^	84^cd^	93^d^	96^d^
Dissolvable	62^a^	100^bc^	93^bc^	100^bcd^	95^bcd^	84^ab^	100^cd^	100^cd^	100^cd^
Hard	10^ab^	5^a^	21^abc^	29^bcd^	47^d^	37^cd^	74^e^	87^e^	86^e^
Juicy	43^ab^	36^a^	75^c^	71^bc^	95^d^	95^cd^	94^d^	100^d^	100^d^
Leafy	10^a^	5^a^	21^ab^	48^bcd^	47^cd^	37^bc^	66^d^	87^e^	89^e^
Lumpy	33^a^	55^ab^	79^bc^	100^d^	95^d^	95^cd^	100^d^	100^d^	96^d^
Pureed	48^a^	4^a^	54^ab^	95^c^	84^c^	79^bc^	87^c^	87^c^	86^c^
Rubbery	14^ab^	5^a^	29^bc^	48^cd^	42^c^	53^cd^	71^de^	84^e^	82^e^
Slippery	10^a^	14^a^	46^b^	8^cd^	71^c^	68^bc^	99^e^	98^e^	93^de^
Soft	24^a^	64^b^	89^c^	95^cd^	95^cd^	100^cd^	99^d^	100^d^	100^cd^
Sticky	62^ab^	64^ab^	50^a^	86^bc^	76^bc^	63^ab^	78^bc^	85^c^	89^c^
Foods with skin	24^a^	50^ab^	68^bc^	62^b^	87^cd^	89^cde^	9^de^	97^de^	100^e^
Combination	29^a^	59^b^	82^bc^	90^cd^	92^cde^	100^de^	99^de^	100^e^	100^de^
Soupy/liquidy	52^a^	68^ab^	89^bc^	100^cd^	100^d^	95^cd^	97^cd^	100^d^	100^cd^

Responses were consolidated to a 2-point scale with 0 = not tried and 1 = tried. Results presented are percentages of tried food textures from probit model of tried food textures. Different superscripts indicate significant differences among the AGs. AG, age group.

More than half of the infants in AG1 had already tried *dissolvable*, *sticky* and *soupy*/*liquidy*. In AG2, more than half of the infants had experienced *lumpy*, *soft*, *foods with skin*, and *combination textures*. The textures *creamy*, *juicy*, and *pureed* were tried by more than half of the children in AG3. In AG4, more than half tried *crispy*, *chewy*, and *slippery*. It was only in AG6 that *rubbery* was tried by >50% of infants. Finally, *hard* and *leafy* were only tried by the majority of infants in AG7.

As textures have to be introduced to be rated for management and refusal, smaller sample sizes were observed for these questions, especially in the smaller age groups. Hence, AG1 and AG2 were merged for the following analyses.

### Role of age on food texture experience management

Table 5 delineates the primary impact of age on texture management. Age groups 1 and 2 were combined for the reporting on the texture management. In general, by the time most textures were introduced they were managed “somewhat easily” to “very easily.” Statistically significant differences among AGs in the reported ease of management of food textures were found in 12 out of 16 textures: *crispy*, *dissolvable*, *juicy*, *leafy*, *lumpy*, *pureed*, *rubbery*, *slippery*, *soft*, *foods with skin*, *combination textures*, and *soupy*/*liquidy*. For example, parents reported that infants were significantly better able to manage *crispy*, *dissolvable*, *juicy*, *leafy*, *lumpy*, *rubbery*, and *slippery*, foods with changes starting at AG1/2 to AG3. No statistically significant differences in reported texture management across AGs were found for *chewy*, *creamy*, *hard*, or *sticky* textures.

**Table 5:** Parental responses to the question: “Tell us how well your child can manage these food textures.”

Food texture	*p*-value	Texture management per age group							
		AG1/2 (4–7 months)	AG3 (8–9 months)	AG4 (10–11 months)	AG5 (12–14 months)	AG (15–17 months)	AG7 (18–23 months)	AG8 (24–30 months)	AG9 (31–36 months)
Crispy	<0.001	3.1^a^	4.4^b^	3.8^ab^	4.0^b^	3.9^ab^	4.4^b^	4.6^b^	4.6^b^
Chewy	0.090	3.0^a^	3.9^a^	3.78^a^	3.8^a^	4.3^a^	4.3^a^	4.2^a^	4.0^a^
Creamy	0.484	4.3^a^	4.6^a^	4.6^a^	4.7^a^	4.9^a^	4.6^a^	4.6^a^	4.8^a^
Dissolvable	<0.001	4.0^a^	4.6^b^	4.5^ab^	4.7^b^	4.4^ab^	4.7^b^	4.8^b^	4.7^b^
Hard	0.110	2.7^a^	3.5^a^	2.8^a^	3.4^a^	3.6^a^	3.5^a^	4.0^a^	4.0^a^
Juicy	<0.001	3.7^a^	4.6^b^	4.3^ab^	4.8^b^	4.4^b^	4.7^b^	4.5^b^	4.5^b^
Leafy	0.015	1.50^a^	4.2^b^	3.8^b^	3.9^b^	3.7^b^	4.1^b^	4.1^b^	3.8^b^
Lumpy	<0.001	3.5^a^	4.2^b^	4.3^b^	4.8^b^	4.4^b^	4.7^b^	4.6^b^	4.4^b^
Pureed	0.007	3.6^a^	4.0^a^	3.9^a^	4.3^a^	4.1^a^	4.5^a^	4.5^a^	4.1^a^
Rubbery	<0.001	1.0^a^	3.5^bc^	2.5^bc^	3.75^bc^	4.3^c^	4.1^c^	4.2^c^	4.3^c^
Slippery	<0.001	2.2^a^	4^bc^	3.3^b^	4.4^c^	3.9^bc^	4.2^bc^	4.6^c^	4.5^c^
Soft	<0.001	3.8^a^	4.3^ab^	4.3^ab^	4.7^bc^	4.3^ab^	4.7^bc^	4.7^bc^	4.9^c^
Sticky	0.491	4.0^a^	4.5^a^	4.3^a^	4.6^a^	4.7^a^	4.3^a^	4.4^a^	4.3^a^
Foods with skin	0.001	3.3^a^	3.9^ab^	3.8^ab^	4.2^b^	3.8^ab^	4.4^b^	4.4^b^	4.5^b^
Combination Texture	0.005	3.7^a^	4.2^ab^	4.4^ab^	4.5^ab^	4.2^ab^	4.5^ab^	4.6^b^	4.4^ab^
Soupy/liquidy	0.024	4.4^a^	4.6^ab^	4.7^ab^	4.8^ab^	4.5^ab^	4.7^ab^	4.8^ab^	4.9^b^

Responses were on a 5-point Likert scale ranging from 1 = very difficult; 2 = somewhat difficult; 3 = neither easy nor difficult; 4 = somewhat easily; and 5 = very easily. Results presented are *F* ratios and *p*-values from one-way analysis of variance model of managing textures with Tukey’s post-hoc test. (NA indicates that less than three participants had introduced the texture in this age group). Different superscripts indicate significant differences among the AGs. AG, age group.

### Texture progression map from food texture experience across AGs

Table 6 reflects on the progression of texture mapped as the analysis of food texture experiences across AGs. The profiles encompass food textures perceived by parents as “very” or “somewhat” easily manageable by their children.

**Table 6:** Texture progression map developed from analysis of food texture experiences across the nine age groups.

	Age group	AG1 (4–5 months)	AG2 (6–7 months)	AG3 (8–9 months)	AG4 (10–11 months)	AG5 (12–14 months)	AG6 (15–17 months)	AG7 (18–23 months)	AG8 (24–30 months)	AG9 (31–36 months)
Management difficulty	Somewhat easy	**Dissolvable**	Dissolvable							
		**Soupy**	Soupy							
		**Sticky**	Sticky	Sticky	Sticky	Sticky	Sticky	Sticky	Sticky	Sticky
			**Combination**	Combination	Combination	Combination	Combination	Combination	Combination	
			**Soft**	Soft	Soft	Soft	Soft	Soft	Soft	Soft
			**With skin**	With skin	With skin					
			**Lumpy**	Lumpy	Lumpy	Lumpy	Lumpy	Lumpy	Lumpy	Lumpy
				**Pureed**	Pureed	Pureed	Pureed	Pureed	Pureed	Pureed
					**Chewy**	Chewy	Chewy	Chewy	Chewy	Chewy
					**Crispy**	Crispy	Crispy	Crispy		
					**Slippery**	Slippery	Slippery	Slippery		
							**Rubbery**	Rubbery	Rubbery	Rubbery
								**Leafy**	Leafy	Leafy
								**Hard**	Hard	Hard
	Very easy			**Creamy**	Creamy	Creamy	Creamy	Creamy	Creamy	Creamy
				**Juicy**	Juicy	Juicy	Juicy	Juicy	Juicy	Juicy
				Dissolvable	Dissolvable	Dissolvable	Dissolvable	Dissolvable	Dissolvable	Dissolvable
				Soupy	Soupy	Soupy	Soupy	Soupy	Soupy	Soupy
						Lumpy	Lumpy	Lumpy	Lumpy	Lumpy
						Soft	Soft	Soft	Soft	Soft
						With skin	With skin	With skin	With skin	With skin
									Crispy	Crispy
									Slippery	Slippery
										Combination

Textures in bold format have been newly introduced to >50% of the age group.

Textures being managed “somewhat easily” in AG1 consist of *dissolvable*, *soupy*, and *sticky*. Newly introduced textures in AG2 included *combination*, *soft*, *foods with skin*, and *lumpy*. Children in AG3 also managed *pureed* “somewhat easily.” “Somewhat easy” textures at AG4 included *chewy*, *crispy*, and *slippery*, with no addition in AG5 and the addition of *rubbery* in AG6. By the older AGs, 7, 8, and 9, all food textures were managed “somewhat easily”.

The texture progression map demonstrated no texture in the “very easily” managed category for infants in AG1 and AG2. *Creamy*, *juicy*, *dissolvable*, and *soupy* were managed “very easily” from AG3 and AG4. Additional texture profiles such as *lumpy*, *soft*, and *with skin* were observed from AG5 to AG7. Further additions in AG8 were *crispy* and *slippery*, while the *combination* was managed “very easily” only in AG9. Overall, textures with a higher proportion of being tried were perceived as easier to handle by children.

### Role of age on refusals of food textures

**Figure 3:**
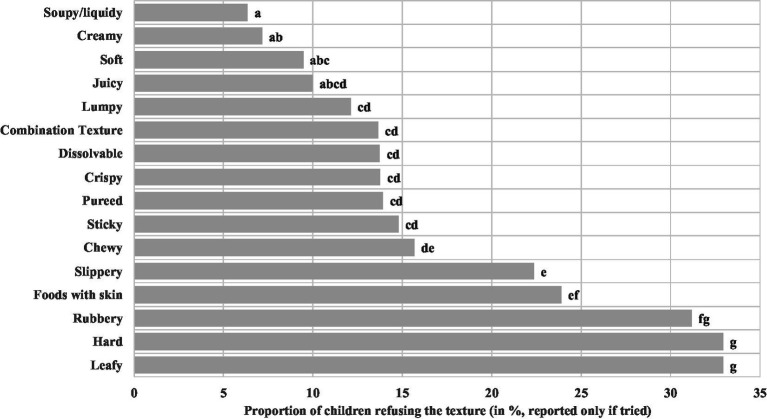
Percentage refusal rate across all age groups. Refusal rate across all age groups, considering only children that tried the texture. Sample size varies from *n* = 167 (*leafy*) to *n* = 291 (*dissolvable*). Different letters indicate significant differences between age groups. Age groups are categorised as follows: AG1 (4–5 months), AG2 (6–7 months), AG3 (8–9 months), AG4 (10–11 months), AG5 (12–14 months), AG6 (15–17 months), AG7 (18–23 months), AG8 (24–30 months), and AG9 (31–36 months).

The proportions of refused food textures per AG are shown in Table 7. Across the AGs, significant differences in refusal of food textures were observed for *juicy*, *lumpy*, *rubbery*, *slippery*, *soft*, and *foods with skin*. The refusal rate for these textures decreased significantly with increasing age. Figure 3 depicts the proportion of refusal rate across all AGs, considering only children that tried the respective textures. Food textures such as *lumpy*, *combination textures*, *dissolvable*, *crispy*, *pureed*, *sticky*, *chewy*, *slippery*, *foods with skin*, *rubbery*, *hard*, and *leafy* were significantly more often refused (proportion of children refusing the textures ranging from 12 to 33%) compared with *soupy*/*liquidy*, *creamy*, *soft*, and *juicy* which were refused by 6–10% of the children. Overall, the proportion of children refusing textures across ages were highest for *hard* and *leafy* (both 33%) followed by *rubbery* (31%), *foods with skin* (24%), and *slippery* (22%). The proportion of children refusing the other textures were substantially smaller, varying from 6% for *soupy*/*liquidy* to 16% for *chewy*. Considering the relatively small number of observations, the actual sample size is provided in Table 7. Additionally, AG1 and 2 have been combined for the reporting on the texture refusal. In AGs1/2 and 3, the most refused food texture was *rubbery*, which was refused by 75% of infants in AG1/2, and 63% in AG3. The refusal rate of foods with skin was 62% in AG1/2. Slippery (59%) was the most commonly refused food in AG4. In AG5, *hard* (44%) was the most refused texture. In AG6, *hard* and *leafy* were both refused by 57% of children. Again, in AG7 *hard* was the most refused food texture (30%), followed by *leafy* with 29%, *slippery*, and *sticky*, both with 28% refusal rate. *Leafy* was the most refused texture (38%) in AG8, while *hard* was the most refused texture in AG9 (42%), followed by *leafy* (36%) and *rubbery* (35%).

**Table 7:** Parental replies to the “Check all that apply” inquiry regarding textures that their children have refused over the preceding month.

Food texture	*z*-score	*p*-value	Texture refusal per age group: number refused/sample size texture introduced (%)							
			AG1/2 (4–7 months)	AG3 (8–9 months)	AG4 (10–11 months)	AG5 (12–14 months)	AG6 (15–17 months)	AG7 (18–23 months)	AG8 (24–30 months)	AG9 (31–36 months)
Crispy	−1.15	0.248	4/14 (29%)	3/13 (23%)	1/12 (8%)	3/28 (11%)	5/13 (38%)	6/59 (10%)	6/60 (10%)	4/27 (15%)
Chewy	0.35	0.727	2/7 (29%)	4/13 (31%)	0/12 (0%)	1/29 (7%)	2/12 (17%)	8/59 (14%)	9/58 (16%)	7/27 (26%)
Creamy	−1.43	0.153	2/11 (18%)	0/14 (0%)	3/14 (21%)	1/29 (3%)	1/14 (7%)	6/57 (11%)	3/57 (5%)	0/27 (0%)
Dissolvable	−0.07	0.942	7/36 (16%)	2/26 (8%)	2/21 (10%)	5/36 (14%)	3/16 (19%)	7/67 (12%)	8/61 (13%)	5/28 (18%)
Hard	−0.63	0.530	1/3 (33%)	2/6 (33%)	2/6 (33%)	8/18 (44%)	4/7 (57%)	15/50 (30%)	13/53 (25%)	10/24 (42%)
Juicy	−2.29	0.021	4/17 (23%)	3/21 (14%)	2/15 (13%)	3/36 (8%)	4/18 (22%)	5/64 (8%)	4/61 (7%)	1/28 (4%)
Leafy	1.68	0.092	0/4 (0%)	0/6 (0%)	2/10 (20%)	7/18 (39%)	4/7 (57%)	13/45 (29%)	20/53 (38%)	9/25 (36%)
Lumpy	−2.14	0.032	8/20 (40%)	3/22 (14%)	2/21 (10%)	4/36 (11%)	3/18 (17%)	3/67 (4%)	4/61 (7%)	6/27 (22%)
Pureed	−1.66	0.098	5/20 (25%)	3/15 (20%)	4/20 (20%)	4/32 (13%)	2/15 (13%)	4/58 (8%)	7/53 (13%)	3/24 (13%)
Rubbery	−2.66	0.008	3/4 (75%)	5/8 (63%)	5/10 (50%)	6/16 (38%)	3/10 (30%)	12/48 (25%)	11/51 (22%)	8/23 (35%)
Slippery	−2.62	0.009	3/6 (50%)	3/13 (23%)	10/17 (59%)	2/27 (7%)	3/13 (23%)	18/66 (28%)	7/60 (12%)	4/26 (15%)
Soft	−2.01	0.044	7/20 (35%)	1/25 (4%)	0/20 (0%)	3/36 (8%)	5/19 (26%)	5/67 (7%)	3/61 (5%)	2/28 (7%)
Sticky	−0.04	0.966	4/27 (15%)	2/14 (14%)	4/18 (22%)	1/29 (3%)	1/12 (8%)	15/52 (28%)	3/52 (6%)	4/25 (16%)
Foods with skin	−4.01	<0.001	10/16 (62%)	7/19 (37%)	4/13 (31%)	8/33 (24%)	7/17 (41%)	10/62 (16%)	9/59 (15%)	4/28 (14%)
Combination Texture	−1.19	0.233	6/20 (30%)	4/23 (17%)	0/19 (0%)	4/31 (11%)	3/19 (16%)	10/66 (16%)	5/61 (8%)	4/28 (14%)
Soupy/liquidy	−0.09	0.924	2/26 (8%)	0/25 (0%)	3/21 (14%)	1/38 (3%)	3/18 (17%)	3/65 (6%)	4/61 (7%)	1/28 (4%)

Outcomes are presented as percentage “yes” responses from the respective tried food textures identified in the survey question “Tell us if your child has refused these food textures” AG = age group.

### Parental concerns about texture introduction

Table 8 shows the main concerns of mothers in terms of introducing new foods to their children. These included expiry (68%), gagging (65%), sharpness of food (64%), choking (63%), and size of food (61%). To address these concerns, 73% of mothers cut food down into small pieces, or crush or blend the food items. The top five safety measures include preparing the foods at home (72%), observing closely when new foods are introduced (72%), cooking food completely (71%), and limiting the amount of food eaten/not allowing self-feeding (69%).

**Table 8:** Safety concerns of Indian mothers when introducing complementary feeding and mitigation measures.

Order	Safety concerns	Cited	Reassurance	Cited	Safety measures	Cited
1	Expired/gone bad	68%	Good expiration date/freshly produced	91%	Cut down food in small pieces/crush/blend	73%
2	Could gag/cough	65%	Trusted brand	90%	Home-made	72%
3	Food is sharp/could cut mouth	64%	Just cooked/hot	86%	Observe closely child (always/for new foods only)	72%
4	Could choke	63%	Well-sealed/tamper-proof package	85%	Cook foods completely/boil	71%
5	Size of food pieces	61%	Ingredients list	63%	Control the amount of food/do not let child feed themselves	69%
6	First time eating food/new food	60%	Bought from trusted store/source	60%	Wash or sanitise plates/cutlery	65%
7	Chemical contamination	60%	On-package positioning	43%	Make sure child washes hands	64%
8	Contains artificial colours/flavours	58%	Organic	32%	Space introduction of new foods to check for allergies or bad reactions	46%
9	Fake/knockoff products	54%			No specific actions, feel confident	45%
10	Food poisoning/bacterial/viral contamination	54%			Took CPR or first aid course	12%
11	Adulterated/tampered with	53%				
12	Contains preservatives	43%				
13	Texture	39%				
14	Nutrition of food	36%				
15	Contains allergens	35%				
16	Contains sugar	33%				
17	Heavy metal contamination	32%				
18	Contains salt	30%				
19	Contains additives	21%				
20	Contains gluten	20%				

## Discussion

This survey aimed to evaluate food texture experiences of children aged 4–36 months, by providing a food texture progression map for an Indian population across nine AGs. To our knowledge, this is the first survey of its kind in India.

The average age for introducing complementary foods in our study was 6.3 ± 1.6 months, aligning closely with the Indian recommendation that children be exclusively breastfed for the first 6 months of life (40). However, this contrasts with reports indicating that many children under 6 months consume other liquids, such as plain water (10%), other milk (8%), or complementary foods (11%) in addition to breast milk; 96% of the study cohort had a history of breastfeeding, either in the past or at present. This is higher than the 62.9–91% reported for Western populations (14, 19). Overall, the proportion of texture-sensitive children in our study was 67%, which is substantially higher than the 23% reported by the US population (22). While the definition of picky eating is broader than texture sensitivity, the two behaviours have commonalities. In India, the proportion of picky eaters at 1 year of age has been reported at a level of 32% (41). The high value of texture-sensitive children in our study might be linked to a low education on the topic of texture introduction and repeated exposure required for acceptance. The mean (SD) age for the introduction of complementary food in this study was 6.3 (1.6) months, which is later than reports from Europe and the United States, where a typical range of 4–6 months is reported (13, 19, 42). However, it does align with recommendations from the Indian Council for Medical Research, which promotes the introduction of complementary food at 6–12 months (43). It could be hypothesised that the higher proportion of breastfeeding and the later onset of introducing complementary food are in part due to cultural practices in India.

### Role of age on food texture introduction and food texture management

In the current study, we divided the study sample into nine AGs, rather than the five groups used in previous studies (19), which provided greater detail on the differences between respective AGs. A guideline aligning the introduction of food textures with specific age groups has been proposed, consisting of narrow intervals of approximately 2 months up to the first year of age (34). However, the split in our study resulted in a slight imbalance in sample size between AGs, with AG7 and eight somewhat overrepresented; still, significant differences were found between age groups and allowed a detailed description of texture introduction in younger age groups (19). However, the subdivision of AGs limits the direct comparison with Surette et al. Even though 4.9 textures were tried in the youngest age group, a rapid increase of textures tried was observed between AG2 (6–7 months) and AG4 (10–11 months), suggesting that the majority of texture introduction occurred before 12 months of age, followed by a slower introduction of the remaining textures between 12 and 36 months. Surette et al. reported a similar pattern, with over 98% of children having tried *creamy*, *dissolvable*, *juicy*, *lumpy*, *and soft* being tried by the age of 12 months (19). However, in our study we found that only 76% of children had tried *creamy* by 12 months.

The finding that across all AGs, textures such as *dissolvable*, *soft* and *soupy*/*liquidy* were tried by most infants, while *rubbery*, *hard,* and *leafy* were tried by substantially fewer infants, corroborates other reports (1, 14, 19). Indeed, dal water and rice water, both of *soupy*/*liquidy* texture, have been reported as the first complementary foods and are characterised by a thin consistency (30). The same study reported dal and rice with porridge/khichdi and chapatti being introduced to 7–8-month-old children, which would fall into the *soft* and *easy-to-chew* category.

The basic foods in India used for complementary feeding belong to cereals, roots, and tubers. These typically include the local staple foods such as rice, wheat, maize, ragi, jowar, and roots (44). In the present study, textures likely to be introduced early (AG1) were *dissolvable*, *sticky*, and *soupy*/*liquidy*, while *hard* and *leafy* tended to be introduced when the children were older (AG7). Biscuits have been reported as a common first food in India (45), and “glucose biscuits” were provided as an example of the dissolvable texture. However, glucose biscuits could be served in a porridge texture after being dissolved in milk or water, and it is not clear whether participants meant that they fed the biscuits to the baby before or after dissolution. The early introduction of *pureed* with 48% of children in AG1is in line with other reports (1, 19); however, in contrast to the US study (19), *pureed* was not tried by 100% of Indian infants but rather varied from 42 to 86% of infants after AG4. We hypothesise that it may be less common to introduce pureed textures due to the lower availability of pureed commercial baby foods in India. This is consistent with a study finding that only 15% of Indian infants consumed commercial baby foods (46). Cultural differences in food texture acceptability also exist within smaller geographical regions such as Europe. A recent review paper reported that a higher proportion of schoolchildren in Northern Europe prefer hard and particle-containing foods than those in Southern Europe. This preference is likely attributed to differences in culinary habits and food selection between countries (47).

In our survey, parents were most likely to feed textures which are perceived to be easy to manage for the children such as *soupy*/*liquidy*, *soft*, and *lumpy*. In fact, few parents rated textures as difficult to manage by the time the texture was introduced. This has also been observed in the US population (19). This may reflect that mothers only introduce new textures when they feel confident that the children can manage them. The least well-managed textures in AG1/2 were *hard*, *leafy*, *rubbery*, and *slippery* and these were also among the least-often introduced textures in these age groups. Statistically significant changes in texture management across AGs were identified for all textures except *chewy*, *creamy*, *hard*, and *sticky.* This finding does not corroborate others who reported significant differences among AGs for the management of 10 food textures (including *chewy*, *hard*, and *soft*) (19). The categorisation of children into eight instead of five AGs may have limited the power of these comparisons. Although not statistically significant for all textures, a trend for the same evolution, i.e., increasing scores, is noticeable for all textures. By AG7, all textures except *hard* were managed easily in our study.

There are no specific recommendations from the Indian Council of Medical Research regarding texture introduction, which, in turn, could result in conservative practices towards food texture introduction (1). Our data revealed that only at 18–23 months a significant proportion of parents (66%, *N* = 20) had introduced *leafy*, although the management of the texture was somewhat easy at already 8–9 months. Considering that age did not significantly impact refusal of the texture *leafy*, exposure at 8 months seems feasible. *Leafy* and *hard* are complex textures that likely support the development of oral skills. Moreover, there is emerging evidence that early introduction of solid foods into an infant’s diet may increase their willingness to eat a variety of fruits and vegetables and decrease their risk of having feeding problems later in life (48). Furthermore, it has been highlighted that consistent and varied food exposure promotes better dietary habits among children (49). The success of repeated exposure strategies is significantly influenced by the type of exposure, as well as parental practices and the food environment. Repeated exposure to specific foods can enhance acceptance not only of those foods but also of other similar foods, underscoring the importance of a diverse and supportive feeding environment (49). For instance, previous studies have observed that parents in the United States and Europe often delay the introduction of lumpy textures in their children’s diets. It is hypothesized that this delay may negatively impact children’s acceptance of a variety of food textures (50). Another hurdle to introducing more complex textures may be explained by the parents’ top concerns in our study, which largely focussed on potential choking or gagging hazards, consistent with commonly reported concerns from other studies (51). Gagging is a protective physiological reaction, and although different from choking, it might be interpreted as choking-related and affect parents’ food choices (20).

### Role of age on refusal of food textures

Texture has been identified as the most commonly refused food characteristic by children (27, 52). Our findings suggest that age plays a significant role in predicting the refusal of only certain textures including *juicy*, *lumpy*, *rubbery*, *slippery*, *soft*, and *foods with skin*. Those refusals tend to decrease with age, consistent with a study where acceptance of *hardness* increased over time from 6 to 18 months (1). The increased acceptance of textures with age may be a reflection of the infants’ improving ability to manage more complex textures and better general oral processing skills that emerge with age (53). On the other hand, not all studies found a decrease in refusal with increasing AG for the *hard* texture (20, 26, 54). In contrast to our findings, Surette et al. (19) reported an increasing refusal probability of nine textures was correlated with increasing age. Hypothetically, the increase in food texture refusals with increasing age could also be due to an increase in the number of children previously exposed to the specific textures or higher rates of picky eating with age (26, 54).

Textures that seem to require a mastering of oral motor skills such as *leafy* and *hard* (the bite force of infants increases with age allowing them to bite harder foods) are more likely rejected and might also show higher rejection rates in younger children (33). It was demonstrated that bite force increases rapidly with age and quadruples from 9 to 36 months (55). As the bite force of children increases with age, it allows them to bite harder foods. Again, it contrasts the report of increased rejection with increasing age (19).

The most refused textures across AGs in our study were *hard* and *leafy*, both demonstrating a 33% refusal rate. However, it cannot be ruled out that other factors such as flavour influence the finding, as it is difficult to completely isolate texture from other sensory components.

It may be hypothesised that the late introduction of textures such as *leafy* and *hard* could delay the development of oral motor skills, as they are textures that seem to challenge oral motor dexterity. For instance, very few parents introduced *leafy* before 8–9 months of age (AG3) and *to only* to 50% of children above 18 months (AG7). *Leafy* textures may be less frequently introduced in India than other cultures, as raw vegetables are rarely consumed. A commonly consumed *leafy* green would be *methi* (Fenugreek leaves), which is consumed blanched and sautéed. The preparation method might not result in a texture as *leafy* as a raw salad leaf. Overall, our findings emphasise that more research is required to identify the optimal age for the introduction of complex textures.

### Study strengths and limitations

Our study demonstrates several strengths. This is the first study that investigated the relationship between age and food texture experience in young children aged 4–36 months in an Indian population.

While this survey has been used before (19), it was developed for the US population. Therefore, we conducted a pilot study to adapt the survey to the cultural context in India before commencing the actual survey. Another strength is that this survey is based on face-to-face interviews of the parents by trained personnel. This approach likely increased the validity of the survey, in contrast to using an online approach (56). For this pilot survey, standardized questionnaires and trained interviewers were used to further enhance the reliability of the results (57, 58). The authors argued that face-to-face interviews can increase the understanding of the questions and keep the participants focussed. Providing local food examples along with the corresponding textures may have also increased understanding (24) and hence the consistency in the responses. While texture terms might be interpreted differently by parents, the current approach made it possible to measure the introduction of specific texture attributes, rather than to have a score of texture exposure. However, the description of the textures by listed food examples did not include an indication of the preparation method. Therefore, a texture term such as *leafy* might be associated with soft, stewed vegetables in India compared to a fresh salad leaf in the United States. Some preparation indication or visuals might provide more detail in the texture definition in future study, similar to the pictograms used by Chow et al. (59).

Moreover, the detail of food texture experience assessed over nine AGs has not been shown before. In the absence of a standardised tool for assessing food acceptance in young children, this texture progression map from food texture experiences across nine AGs in young children represents a reflection of current practices in an Indian population and may provide guidance for future research on the introduction of food textures.

A limitation of this study is the lack of broad representativeness. The survey was conducted in a relatively small sample size in urban areas, which limits the generalizability of the results (13). In addition, as dietary habits and dietary quality can vary between urban vs. rural areas (60, 61), the focus on urban areas may have influenced the current analysis. Furthermore, aspects such as parents’ income, accessibility, convenience, or behavioural influence may also affect parents’ choices when introducing complementary foods to young children (61, 62). While the right-hand rule can offer simplicity, consistency, and efficiency, it might introduce bias if there are systematic differences between households or individuals on the right side versus the left side of the street. The current study included limited data about accessibility, convenience, and demographic information of the parents, especially socioeconomic data would help the understanding of the influence of demographic factors on food texture experience (63). Although this study used face-to-face interviews with trained personnel, a potential for bias in responses or misunderstanding of questions about when textures are introduced or refused cannot be ruled out. However, two recent studies using parental recall of complementary feeding practices found consistent results across samples, suggesting that parental recall was an effective way to measure these behaviours (64, 65). However, the use of parental reports in a cross-sectional design did not allow to follow the development of the same cohort of children at all ages and a longitudinal study would have provided more robust data. A more solid framework for aligning food texture introduction with developmental readiness has recently been suggested (34). The current survey did not collect data on parents’ feeding practices, which also may have impacted texture exposure and acceptance (66, 67). No questions were included in this study to ascertain whether parents have received advice from a healthcare professional regarding complementary feeding, which could have influenced the age of texture introduction (20). Current methods for studying texture preferences rely primarily on self-reported measures, which raise concerns about their validity and reliability (47). For our study we had a local expert panel review and validate the questionnaire content. Additionally, we avoided leading questions and minimised social desirability bias by guaranteeing anonymity and confidentiality. Still, there remains a need to develop more robust and relevant test tools for studying texture acceptance in children.

In a cultural context, our study found a higher proportion of breastfed children in India than Western populations. Additionally, the proportion of texture-sensitive children in our sample was significantly higher than that reported in the US population (19). Moreover, our data indicate that the average age for introducing complementary foods to Indian children was later than in Europe or the United States. This finding is consistent with a report indicating that North American parents tend to introduce solid foods earlier, whereas in Asia, this introduction is often delayed (50). Cultural factors such as dietary habits, traditional foods, feeding practices, and parental influence are crucial considerations when designing studies to explore food texture exposure. These factors shape children’s food preferences and acceptance, highlighting the need for culturally sensitive approaches in research (47). Understanding these influences and variability underscores the necessity for region-specific guidelines to ensure culturally appropriate feeding practices.

Our texture progression map can serve as a guide to determine when children might be ready for specific textures. This map can also inform parents about the diverse range of textures available to expose their children to, promoting varied and balanced dietary experiences. Furthermore, as most parents reported that textures were easy or somewhat easy once introduced, it suggests that children might be ready for new textures earlier than parents typically expect. These practical insights aim to support parents and healthcare professionals in fostering better dietary habits in children. However, due to the limitations of this initial study, we refrain from making overly bold claims and recommend further research to confirm these findings. Future research should consider increased sample size, broader recruitment strategies, texture sensitivity reporting, and including demographic data in analysis to further strengthen the understanding of differences in food texture exposure and acceptance between AGs and increase generalizability.

## Conclusion

This study offers fresh insights into the acceptance of food textures among infants aged 4–36 months in India, confirming that acceptance of certain food textures tends to develop with age. The granularity of the findings was enhanced by categorising children into nine age groups. *Dissolvable*, *sticky*, and *soupy*/*liquidy* textures were already accepted by more than half of 4–5-month-old infants. Interestingly, *soupy*/*liquidy* in India is a more common base texture than *pureed*, the latter being more prevalent case for the EU and the United States. In our study, *pureed*, often the first texture introduced when commencing complementary feeding in other countries, was introduced to a majority of infants from age group 3 (8–9 months) onwards. Food textures that seem to require more developed oral motor skills, such as *rubbery*, *slippery*, and *foods with skin*, are more likely rejected and might be proportionally more frequently rejected in younger children.

As found by others, refusal tends to decrease with the ability of the infants to manage texture as acceptance increases with the maturing of oral motor skills.

The differences in age of complementary food introduction and the role of age on acceptance of certain textures in this study compared with data from other countries point to cultural differences and therefore highlight the necessity to generate more country-specific evidence around texture introduction. In general, the potential biases inherent in this type of survey warrant caution when interpreting the results. Future research would benefit from investigating the link between child developmental skills, such as motor skills, and readiness for different textures, and how this relates to self-feeding and cultural practices.

This study emphasises the importance of introducing a variety of textures in this critical period of children’s development. Based on our data, the management of textures was relatively easy for children as young as 8–9 months. Therefore, introducing textured foods at this age could be suggested. Future development of more specific guidelines for parents and healthcare professionals on the progression of introducing textures to infants’ diets is needed.

## Data availability statement

The raw data supporting the conclusions of this article will be made available by the authors, without undue reservation.

## Ethics statement

The requirement of ethical approval for the studies involving humans was waived by Institutional Review Board of Washington State University (IRB #17585). The studies were conducted in accordance with the local legislation and institutional requirements. The participants provided their written informed consent to participate in this study.

## Author contributions

MD: Data curation, Formal analysis, Methodology, Project administration, Visualization, Writing – original draft, Writing – review & editing. FT: Writing – original draft, Writing – review & editing. NA: Data curation, Formal analysis, Methodology, Visualization, Writing – review & editing. LF: Conceptualization, Methodology, Writing – review & editing. CR: Conceptualization, Methodology, Writing – review & editing. SS-S: Conceptualization, Data curation, Funding acquisition, Investigation, Methodology, Supervision, Validation, Writing – review & editing.
