# Supplementary material for: Food texture experiences across nine age groups in Indian infants from urban areas
Source: Front Nutr. 2024 Jul 18;11:1419718. doi: 10.3389/fnut.2024.1419718 (PMC11292795; doi:10.3389/fnut.2024.1419718)
Supplement: Supplementary file 1 [file Table_1.DOCX]

# Supplementary material

Supplementary table 1 Reported education of mothers per recruitment center

| **Education** | **Kolkata** | **Delhi** |
| --- | --- | --- |
| Incomplete Secondary Education | 16 | 3 |
| Secondary Education Completed | 66 | 74 |
| Some University or Vocational Certification | 10 | 9 |
| Vocational or Professional Certification Completed | 7 | 3 |
| University Education Completed | 29 | 45 |
| Postgraduate Education Completed | 21 | 21 |
| Doctorate, Post-doctorate or equivalent Completed | 2 | 0 |
| **Total** | **151** | **155** |

Supplementary table 2 Reported income of household per recruitment center

| **Income** | **Kolkata** | **Delhi** |
| --- | --- | --- |
| Under ₹ 1,00,000 | 11 | 13 |
| ₹ 1,00,000 to ₹ 4,99,999 | 88 | 103 |
| ₹ 5,00,000 to ₹ 9,99,999 | 19 | 18 |
| ₹ 10,00,000 to ₹ 14,99,999 | 13 | 17 |
| ₹ 15,00,000 to ₹ 19,99,999 | 9 | 4 |
| ₹ 20,00,000 to ₹ 29,99,999 | 2 | 0 |
| ₹ 50,00,000 or more | 1 | 0 |
| Prefer not to answer | 8 | 0 |
| **Total** | **151** | **155** |
